# Understanding the Mechanism of Action of NAI-112, a Lanthipeptide with Potent Antinociceptive Activity

**DOI:** 10.3390/molecules26226764

**Published:** 2021-11-09

**Authors:** Arianna Tocchetti, Marianna Iorio, Zeeshan Hamid, Andrea Armirotti, Angelo Reggiani, Stefano Donadio

**Affiliations:** 1 Naicons Srl, Viale Ortles 22/4, 20139 Milan, Italy; atocchetti@naicons.com (A.T.); sdonadio@naicons.com (S.D.); 2 D3 Validation, Fondazione Istituto Italiano di Tecnologia, Via Morego 30, 16163 Genoa, Italy; bio.zeeshan@gmail.com (Z.H.); angelo.reggiani@iit.it (A.R.); 3 Analytical Chemistry Lab, Fondazione Istituto Italiano di Tecnologia, Via Morego 30, 16163 Genoa, Italy; andrea.armirotti@iit.it

**Keywords:** lanthipeptide, untargeted lipidomics, lysophosphatidic acid, TPRV1, lipid II, VISA strains

## Abstract

NAI-112, a glycosylated, labionine-containing lanthipeptide with weak antibacterial activity, has demonstrated analgesic activity in relevant mouse models of nociceptive and neuropathic pain. However, the mechanism(s) through which NAI-112 exerts its analgesic and antibacterial activities is not known. In this study, we analyzed changes in the spinal cord lipidome resulting from treatment with NAI-112 of naive and in-pain mice. Notably, NAI-112 led to an increase in phosphatidic acid levels in both no-pain and pain models and to a decrease in lysophosphatidic acid levels in the pain model only. We also showed that NAI-112 can form complexes with dipalmitoyl-phosphatidic acid and that *Staphylococcus aureus* can become resistant to NAI-112 through serial passages at sub-inhibitory concentrations of the compound. The resulting resistant mutants were phenotypically and genotypically related to vancomycin-insensitive *S. aureus* strains, suggesting that NAI-112 binds to the peptidoglycan intermediate lipid II. Altogether, our results suggest that NAI-112 binds to phosphate-containing lipids and blocks pain sensation by decreasing levels of lysophosphatidic acid in the TRPV1 pathway.

## 1. Introduction

Ribosomally synthesized and post-translationally modified peptides, or RiPPs, form a diverse group of natural products characterized by a peptide skeleton that can undergo a number of post-translational modifications [1]. In the past 20 years, new members have been added to this class of secondary metabolites, mostly thanks to the development of genome-mining tools to detect RiPP biosynthetic gene clusters in the growing microbial genome databases. Over 20 different RiPP families are known, each carrying unique chemical features [2]. This high structural diversity is due to the various post-translational modifications that impart new chemical functionalities on the precursor peptides, leading to core peptides that carry multiple variable sites [3]. In some cases, the discovery of a new RiPP has been accompanied by establishing the correspondent bioactivity [1,3]. However, because most new RiPPs are being discovered through approaches based on structural novelty, they are mostly orphan of an associated biological function (e.g., [4,5]) unless they have measurable antimicrobial activity.

**Figure 1:**
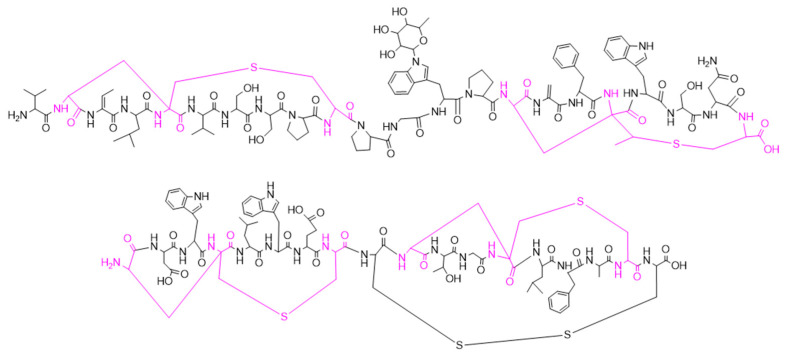
Structures of NAI-112 (**upper**) and labyrinthopeptin A2 (**lower**). In pink, the amino acids involved in the labionin bridges are highlighted.

NAI-112 is a labionine (Lab)-containing lanthipeptide with a glycosylated tryptophan residue and an unusual methyl-Lab bridge [6]. It was discovered in a phenotypic screening program for inhibitors of bacterial cell wall biosynthesis. It showed modest antibacterial activity with a minimal inhibitory concentration (MIC) of 32 μg/mL against *Staphylococcus aureus*. At the time of its discovery, there was only one precedent of Lab-containing lanthipeptides, the labyrinthopeptins [7]. In particular, despite having unrelated amino acid sequences, NAI-112 and labyrinthopeptin A2 share a similar ring topology, with identically sized rings A and A’ and similarly sized rings B and B’ (Figure 1). A longer loop separates B and A’ rings in NAI-112 than in labyrithopeptin A2. The latter molecule was shown to have moderate antiviral activity but was highly active in a neuropathic pain model [7]. Intriguingly, we found that also NAI-112 shows analgesic activity. It was active on both nociceptive and neuropathic pain, with good responses in the formalin test (early and late phases) and in the chronic constriction injury of the sciatic nerve model [6].

In mammals, many different pathways can lead to pain sensation and the inhibition of many of them has been exploited by marketed drugs [8]. Knowledge about the pathway can properly direct the development path of a new drug candidate and address potential side effects [9]. For these reasons, it is important to establish the mechanism(s) of the analgesic activity of NAI-112 to evaluate its potential as a drug candidate. As a step in that direction, we report here the result from lipidomic studies in mice and the characterization of resistant mutants of *S. aureus*. Overall, our results are consistent with NAI-112 binding to lipids and interfering with the TPRV1 pathway.

## 2. Results

### 2.1. Lipidome Analysis in the Spinal Cord

**Figure 2:**
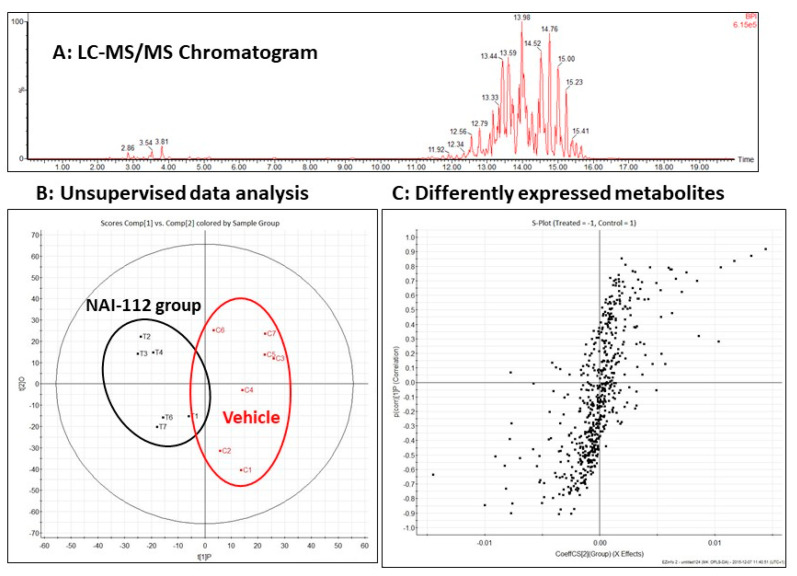
Lipidomic study. (**A**) Representative chromatogram of HR-LC-MS analysis of untargeted lipidomics. (**B**) Feature clustering using principle component analysis (PCA). Vehicle group in red shows clear separation from the NAI-112-treated group. (**C**) Orthogonal projection to latent structures–discriminant analysis (OPLS-DA) was used to identify lipids differentially expressed between vehicle and NAI-112 groups.

**Figure 3:**
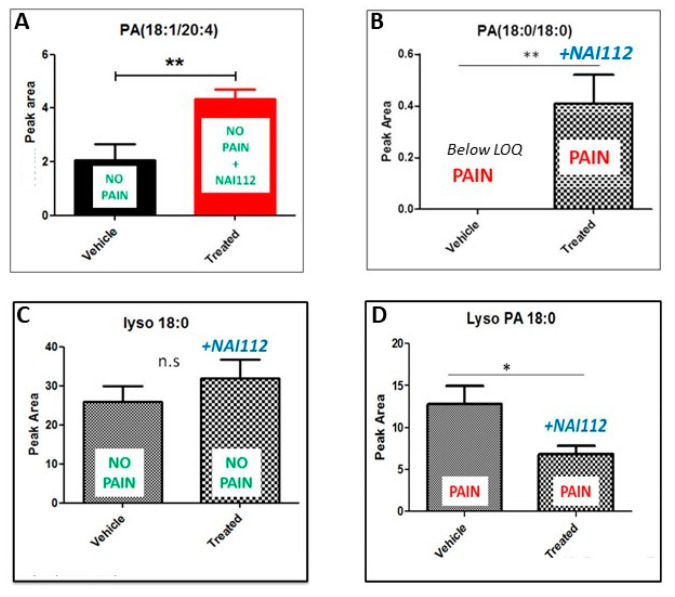
Effect on levels of phosphatidic acid (PA) and lysophosphatidic acid (Lyso PA or LPA). The levels of PA (18:1/20:4) and PA (18:0/18:0) increased by NAI-112 treatment in both naive and in-pain mice (**A**,**B**). No significant effects were observed on LPA levels in no-pain mice (**C**), while LPA (18:0) reduced after treatment with NAI-112 in in-pain mice (**D**). In all panels, values are expressed as means ± SEM. The two-tailed t-test was used to assess statistical significance: * *p* < 0.05; ** *p* < 0.01; n.s., not significant.

To understand the effect of NAI-112 and learn its possible mechanism in nociception, we performed an untargeted lipidomics experiment using mice spinal cords to observe any possible differential lipids that might play a role in NAI-112 nociception. We initially compared two experimental groups (vehicle *n* = 7 and NAI-112 group *n* = 6), where mice were treated with 30 mg/kg body weight of NAI-112 using IP injection and spinal cord samples were collected after 2 h for lipid extraction. After total lipid extraction from the samples, we acquired high-resolution LC-MS/MS data. A representative chromatogram of the mass-spectrometer-based untargeted lipidomics analysis is shown in Figure 2A. After data acquisition, we used dedicated statistical tools for analyzing the possible differential lipids between experimental groups. As shown in Figure 2B, using principle component analysis (PCA), we observed clear separation between experimental groups, and we then selected differentially expressed lipids using orthogonal projection to latent structures–discriminant analysis (OPLS-DA), as shown in Figure 2C. Among other significantly altered lipids, we observed a significantly sharp increase in the levels of phosphatic acid (18:1/20:4, 1-oleoyl-2-eicsoatetraenoyl-PA), as shown in Figure 3A.

There are several reports showing a role of phosphatic acids (PAs) and of their hydrolytic product lysophosphatidic acids (LPAs) in pain sensation. Neuropathic pain damage to the nerve along the pain pathway results in spontaneous generation of action potential and lowered nociceptive threshold, as seen in allodynia and hyperalgesia. This abnormal pain transmission had been linked to LPA production in the spinal cord [10,11,12]. Having observed an effect of NAI-112 on PA levels in naive mice, we wanted to see what happens in mice when pain is induced before NAI-112 injection. So we re-performed the untargeted lipidomics experiment using three experimental groups using seven mice for each group: vehicle, pain model (sciatic nerve ligation model [6]), and treated (pain model + NAI-112). The lipidomics experimental workflow and data analysis remained the same, but we majorly focused on PAs and LPAs and then overlapped the two experimental results (with and without pain). As shown in Figure 3, the level of PA (18:1/20:4) and PA (18:0/18:0, distearoyl-PA) increased in the presence of NAI-112 in both naive and in-pain mice (panel A and B). We then looked at the LPA levels across groups. No significant effects were observed on LPA levels in no-pain mice (panel C), while the level of LPA (18:0, stearoyl-PA) was significantly reduced in the presence of NAI-112 in in-pain mice (panel D).

Overall, treatment with NAI-112 did not lead to profound alterations in the spinal cord lipidome but led to an increase in PA levels and a concomitant decrease in LPA levels. Interestingly, LPA has been reported as a chemical signature of neuropathic pain since it activates the TRPV1 receptor, triggering pain sensation [10,11].

### 2.2. Effects on Enzymes in the TPRV1 Pathway

The lipidome data are consistent with an effect on the TRPV1 pathway and suggest that NAI-112 directly or indirectly interferes with the formation of LPA. A previous experiment carried out using NAI-112 and a selected panel of pain-related receptors (Ca^2+^ channel, norepinephrine transporter, TRPM8, and TRPV1) showed no antagonist effect of NAI-112 by direct binding to TRPV1 (see the Materials and Methods section for details) We thus investigated whether NAI-112 can affect any of the enzymes acting upstream of TRPV1 according to the model proposed by the Nobel Prize winner David Julius and coworkers [13]. Using commercially available kits, we unexpectedly observed that NAI-112 inhibits multiple enzymes of the pathway, such as protein kinase C and several phospholipases. Oddly, the activity of some of the enzymes was enhanced by low NAI-112 concentrations and the inhibition curves did not show the expected sigmoid shape. Of note, all these enzymes require micelles for optimal activity and were inhibited by NAI-112 with similar IC_50_s (Supplementary Figure S1). A possible explanation for the above observations is that NAI-112 strongly interacts with micelles, enhancing their stimulatory effect at low concentrations and then disrupting their structure in a linear concentration-dependent manner, thus interfering with the activity of micelle-requiring enzymes. The above results are consistent with the possibility that NAI-112 directly binds PAs, sequestering them from processing by phospholipases and thus leading to decreased LPAs in the spinal cord.

### 2.3. Binding Experiments

**Figure 4:**
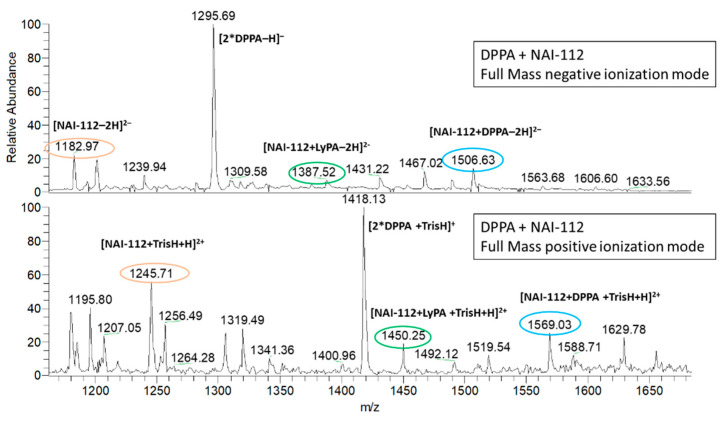
Mass spectra of the sample containing DPPA and NAI-112 in negative ionization mode (**upper** spectrum) and in positive ionization mode (**lower** spectrum). Addition of Tris to improve DPPA solubility led to the formation of Tris adducts, visible in positive ionization mode. The orange circle indicates the lanthipeptide alone, and green and blue circles show the lanthipeptides complexed with LPA and DPPA, respectively.

We thus tested whether NAI-112 is able to form a complex with either PAs or phosphatidylethanolamines (PEs). When 0.4 mM 1,2-dipalmitoyl-phosphatidic acid (DPPA) or 1,2-dipalmitoyl-phosphatidylethanolamine (DPPE) was incubated with an equimolar amount of NAI-112 for 30 min at room temperature and analyzed by direct infusion in a mass spectrometer in negative and positive ionization modes, after 10-fold dilution with 50% acetonitrile, a signal at *m/z* 1506.6 [M − 2H]^2−^ was observed in the binding mixture containing NAI-112 and DPPA (Figure 4). A corresponding signal at *m*/*z* 1569 [M + TrisH + H]^2+^ was observed in positive ionization mode (Figure 4). Isolation of the signal and fragmentation confirmed its identity with a 1:1 complex of DPPA and NAI-112 (Supplementary Figure S2A). In contrast, a complex between NAI-112 and DPPE, a lipid carrying a positive charge, could be observed only in trace amounts (Supplementary Figure S2B). 

When performing these studies, we realized that the commercial DPPA sample contains a detectable amount of LPA. By zooming in the range between *m*/*z* 1300–1800 both in negative and in positive ionization mode in the DPPA-NAI112 sample, a signal at *m*/*z* 1387.7 [M-2H]^2−^ was detected, consistent with a 1:1 complex of NAI-112 with LPA (Figure 4).

Altogether, these results suggest that NAI-112 can form complexes with mono- or dipalmitoyl-glycerol with an unmodified phosphate group.

### 2.4. Isolation of NAI-112-Resistant Mutants

NAI-112 was discovered in the course of a screening program for peptidoglycan biosynthesis inhibitors and showed modest inhibitory activity against staphylococci and streptococci [6]. We reasoned that if the antinociceptive activity of NAI-112 is due to binding to lipid components, a similar mechanism might be responsible for its antibacterial activity. Thus, isolation of bacterial strains resistant to NAI-112 might shed light on its molecular target. We thus decided to look for NAI-112-resistant mutants by direct selection on media containing NAI-112 and by serial passages in the presence of sub-inhibitory concentrations.

When *S. aureus* cells were plated in NAI-112-containing medium, no spontaneous resistant mutants were obtained in the presence of 160 or 640 μg/mL of NAI-112. Thus, at these concentrations (equivalent to 5× and 20× its MIC, as determined in liquid medium), spontaneous resistant mutants occur at a frequency of <10^−9^ CFU/mL. No attempts were made to select resistant mutants at lower NAI-112 concentrations.

**Figure 5:**
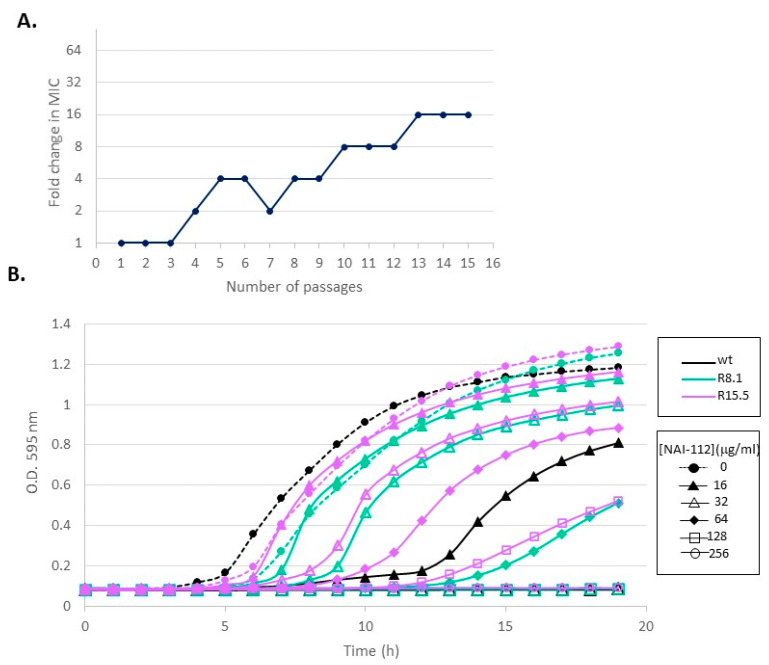
NAI-112-resistant mutants. (**A**) Relative change in NAI-112 MIC after serial passages of *S. aureus* ATCC 6538P. The starting MIC was 32 µg/mL. (**B**) Growth of the wild type (wt) and two mutants selected after the 8th (R8.1) and the 15th (R15.5) passage from the experiment in panel (**A**). See the Isolation of NAI-112-Resistant Mutants section for details.

We were, however, able to generate resistant strains by serial passages in the presence of sub-inhibitory concentrations of NAI-112. In general, multiple passages at the same sub-inhibitory concentration were required before *S. aureus* was able grow at the next higher concentration. After the eighth passage, the strain was able to grow at 64 μg/mL of NAI-112, and after the fifteenth passage, growth was observed in a culture containing 256 μg/mL of NAI-112 (Figure 5A).

From the population growing at the highest NAI-112 concentrations after passages 8 and 15 (64 and 256 µg/mL, respectively), single colonies were isolated after plating in antibiotic free-medium. Two colonies from the 8th passage (designated R8.1 and R8.2) and five from the 15th passage (designated R15.1 through R15.5) were analyzed for growth and antibiotic susceptibility. An example for colonies R8.1 and R15.5 is shown in Figure 5B. In the absence of NAI-112, the two mutant strains and the wild type grew at a similar rate. However, while growth of the wild type was retarded and fully inhibited at 16 and 32 µg/mL of NAI-112, respectively, 64 and 128 μg/mL of NAI-112 was required to observe a similar effect on mutant strain R8.1. Mutant strain R15.5 was slightly more resistant, showing reduced growth at 128 µg/mL and no growth at 256 µg/mL. The growth curves of the other analyzed mutant strains are shown in Supplementary Figure S3. Similarly, mutant strain R8.2 showed reduced growth and no growth at 64 and 128 µg/mL, respectively, while mutant strains R15.1–R15.4 behaved similarly, with growth retardation and growth inhibition observed at 128 and 256 µg/mL of NAI-112, respectively. In summary, the analysis of individual colonies confirmed the results from population studies (Figure 5A), indicating that the strain had become increasingly resistant to NAI-112 through serial passages.

We then wondered whether any of the mutant strains had become resistant to other antibiotics in addition to NAI-112. The results for mutant strains R8.1 and R15.5 are reported in Table 1. Interestingly, there was a modest but consistent shift in the MICs of vancomycin, ramoplanin, and NAI-107 against R8.1 and R15.5. These antibiotics all bind to the essential peptidoglycan precursor lipid II and are known to be partially affected by the mutations arising in the so-called vancomycin-insensitive *Staphylococcus aureus* (VISA) strains [14,15]. In contrast, the MICs of erythromycin, ciprofloxacin, and rifampicin, which target other cellular processes, were not affected.

### 2.5. Genome Analysis of Resistant Strains

**Figure 6:**
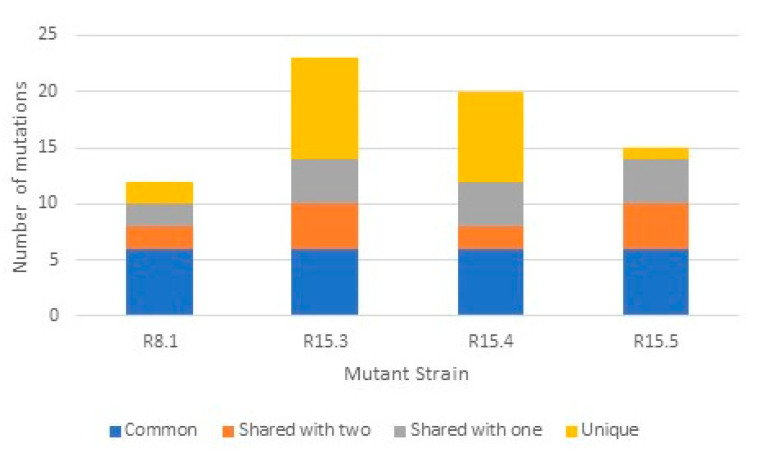
Number of mutations (SNPs or INDELs) observed in each mutant strain in comparison with the parental strain and their distribution in comparison with the other mutants.

To identify the mutations responsible for NAI-112 resistance, we compared the genomes of the parental strain and of the mutants R8.1, R15.3, R15.4, and R15.5. This analysis led to the identification of 37 mutations, including 33 single-nucleotide polymorphisms (SNPs) and 4 insertion or deletion of bases (INDELs). Six SNPs were common to the four mutants. The remaining SNPs/INDELs were distributed, as shown in Figure 6, with the majority of mutations shared by at least two mutants. All mutant strains carry different mutations, so they are not siblings. The SNPs observed in at least two mutants are listed in Table 2, while the remaining 20 unique mutations are reported in Supplementary Table S1. (Notice that 16 of the 37 SNPs with respect to the parental strain matched the corresponding sequences in the deposited ATCC 6538P genome.)

Of the six SNPs common to the four mutants, only one led to an amino acid change in the corresponding gene product, Cys598Tyr in WalK (Table 2), a two-component-system sensor kinase involved in cell wall metabolism and previously reported as a necessary but not sufficient mutation to conferring decreased susceptibility in some VISA lineages [18,19,20]. The other five common SNPs fall in intergenic regions or represent synonymous substitutions in transposase genes, suggesting they affect gene expression. Interestingly, four mutations fall within SAFDA_1386, which belongs to the DUF1672 family, a major component in the *S. aureus* lipoproteome [21]: two as synonymous substitutions and two that result in changes of two distinct amino acids (Table 2). The other SNPs common to at least two mutants are listed in Table 2.

All together, these results indicate that strains selected for resistance to NAI-112 are phenotypically and genetically similar to VISA strains, which can arise through multiple mechanisms [22,23], one represented by mutations in *walK* [18,19].

## 3. Discussion

The results from the lipidomic experiments in mice, from the binding assay analyzed in MS and from the analysis of the *S. aureus* mutants resistant to NAI-112, are consistent with the hypothesis that NAI-112 binds to one or more phosphate-containing lipids and, by doing so, interferes with the processing of these lipids by relevant enzymes. In mice, this interference translates in lower LPA levels. It has been reported that if in control mice, LPA produces acute pain-like behaviors, those effects are substantially reduced in *Trpv1*-null animals. It was demonstrated that TRPV1 is a direct molecular target of the pain-producing molecule LPA, and that is the first example of LPA binding directly to an ion channel to acutely regulate its function [24]. Since LPA has been reported to activate the TRPV1 receptor triggering pain sensation, a decrease in LPA is expected to result in pain relief. That NAI-112 acts in the TRPV1 pathway is also consistent with previous experiments that demonstrated that pain amelioration by NAI-112 is reverted by the TRPV1 antagonist AMG9810, while NAI-112 does not bind directly to TRPV1 [6].

A few studies have identified the molecular targets of lanthipeptides. Notably, class I lanthipeptides, including nisin, gallidermin, and NAI-107, have been shown to bind to lipid II, followed by pore formation in the membrane or membrane disruption [25,26,27]. Class II lantibiotics, such as mersacidin and actagardin, also bind to lipid II [28]. Interestingly, some structurally unrelated class II lantibiotics, namely cinnamycin and its variants duramycin and ancovenin, have been shown to bind to phosphatidylethanolamine-containing lipids [29,30,31,32]. Two-peptide lantibiotics, exemplified by lacticin 3147, also form a complex with lipid II through their mersacidin-like component [33]. Recently, Medeiros-Silva et al. [34] demonstrated binding of nisin to the pyrophosphate cage of lipid II in membranes of bacterial cells.

While this work was in progress, Prochnow et al. [35] reported that labyrinthopeptins induce a virolytic effect through binding to phosphatidylethanolamine, a component of the viral membrane. Given all these precedents for lanthipeptides, the similar ring topology between NAI-112 and labyrinthopeptins (Figure 1), and the results presented herein, it is tempting to speculate that lanthipeptides are generally able to bind to (pyro)phosphate-containing lipids, and small differences in their structures can result in preferential affinity for various phospholipids. Since only a portion of a lanthipeptide is involved in phospholipid binding, its biological activity might ultimately depend on a combination of the preferred phospholipid and the interaction of the remaining lanthipeptide portion with membrane-associated cellular components.

## 4. Materials and Methods

### 4.1. NAI-112 Source

NAI-112 was obtained after cultivation of *Actinoplanes* sp. DSM24059 in two 4 L bioreactors, followed by purification of the compound according to the procedures described by Iorio et al. [6]. A single batch of product was used for all the experiments described herein. For each study, NAI-112 was freshly dissolved in dimethyl sulfoxide at 10 mg/mL and diluted with the appropriate medium just before use.

### 4.2. Mice Study

Male CD1 mice weighing 25–30 g (Charles River) were used in accordance with the ethical guidelines of the International Association for the Study of Pain and in compliance with Italian and European Economic Community regulations (D.M. 116192; O.J. of E.C. L 358/1 12/18/1986). Mice were housed in groups of 5 or 4 in ventilated cages containing autoclaved cellulose paper as nesting material, with free access to food and water. They were maintained under a 12/12 h light/dark cycle (lights on at 08:00 a.m.) at controlled temperature (21 ± 1 °C) and relative humidity (55 ± 10%).

Sciatic nerve ligations were performed, as described by Iorio et al. [6], and the code number of the authorized animal protocol is Decreto ministeriale n° 41/2010-B. The vehicle or test compound was dissolved in 0.9% sterile saline/5% PEG-400/5% Tween-80 and injected subcutaneously. Spinal cord dissection was done according to Henriques et al. [36]. Mice were sacrificed by decapitation, and spinal cords were rapidly dissected, frozen in liquid nitrogen, and stored at −80 °C until further processing.

### 4.3. Untargeted Lipidomics Sample Preparation

For lipid extraction from spinal cord samples, the Bligh–Dyer protocol was followed. Briefly, equal amounts of samples were dissolved in 2 mL of 2:1 (*v*/*v*) methanol:chloroform, followed by homogenization, vortexing. and sequential addition of 0.6 mL of chloroform and water. Samples were centrifuged at 3500 rpm for 20 min, and the lower organic phase was transferred into clean 4 mL glass vials and dried under a nitrogen stream. Dried samples were reconstituted in 0.4 mL of 9:1 (*v*/*v*) methanol:chloroform.

### 4.4. Mass Spectrometer Data Acquisition

Four microliters of each sample was injected in a Waters UPLC Acquity system coupled to a Synapt G2 QToF high-resolution mass spectrometer operating in negative (ESI-) ion mode. After sample injection, lipids were separated on a reversed-phase C18 CSH column (2.1 × 100 mm) using mobile phase A (10 mM ammonium formate in 60:40 acetonitrile/water) and mobile phase B (10 mM ammonium formate in 90:10 isopropyl alcohol/acetonitrile). The total run time for each sample was 25 min, and the following gradient program was used: 30% mobile phase B for 1 min, which was brought up to 35% in 3 min, then to 50% in 1 min, and then to 100% in 13 min, and then a 1 min isocratic step at 100% mobile phase B, followed by reconditioning to initial conditions until a 25 min total run time. Across the lipid separation, the column temperature was maintained at 55 °C.

For mass spectrometry data acquisition, the following parameters were used: capillary voltages were set at 2 kV and cone voltage at 35 V; the source temperature was 120 °C; desolvation gas and cone gas (N_2_) flows were 800 L/h and 20 L/h, respectively; and the desolvation temperature was set to 400 °C. Data were acquired in MSe mode and MS/MS fragmentation performed in the trap region. Low-energy scans were acquired at fixed 4 eV potential, and high-energy scans were acquired with an energy ramp from 25 to 45 eV. The scan rate was set to 0.3 s per spectrum. The scan range was set to 50 to 1200 *m*/*z*. Leucine enkephalin (2 ng/mL) was infused as a lock mass for spectra recalibration.

### 4.5. Multivariate Data Analysis and Lipid Identification

After data acquisition, raw data files were loaded on MarkerView™ Software (Applied Biosystems/MDS Sciex, Toronto, Canada) to generate principle component analysis (PCA) and orthogonal projection to latent structures–discriminant analysis (OPLS-DA) plots for generation of differential lipids across groups. Analytes were identified using Metlin and HMDB databases with a mass tolerance of 5 ppm. The accurate mass list was searched against the METLIN [37] and HMDB [38] web-based metabolomics algorithms, with strict *m*/*z* tolerance of 5 ppm and allowing [M − H]^−^, [M + FA − H]^−^ and [M − H_2_O − H]^−^ as adducted species for negative ion mode. Criteria for matching included accurate mass matching, class specific retention time. and adduct type consistency, as reported by Cajka et al. [39], in addition to indications on MS/MS fragmentation patterns already available in the literature [40].

GraphPad Prism software was used for final visualization of significant lipids and calculation of *p*-values.

### 4.6. Pain-Related Receptor Antagonist Effect

Experiments for the evaluation of the antagonist effect of NAI-112 versus a selected panel of pain-related receptors were carried out by Eurofins (studies n. 100004631 and 100003623, www.eurofins.com, accessed on 28 September 2012). In particular, NAI-112 at a concentration of 10^−5^ M was assayed with a Ca^2+^ channel, norepinephrine transporter, TRPM8, and TRPV1, using omega-conotoxin GVIA, protriptyline, 4-(3-chloro-2-pyridinyl)-N-[4-(1,1-dimethylethyl)phenyl]-1-piperazinecarboxamide (BCTC), and capsazepin as positive controls, respectively.

### 4.7. Enzymatic Assays

To access NAI-112 effects on enzymatic activities involved in the vanilloid-sensitive pathway, commercially available kits were used. Calibration curves to determine the enzyme concentration for the assay were performed, and all assays were run following the instructions provided in the commercial kits. All experiments were run in duplicate. Control reactions consisted of a negative control without an enzyme and a positive control in which no inhibitor was added.

For protein kinase C (PKC), we used the ab139437 PKC Kinase Activity Assay Kit (Abcam, UK), which is based on recognition by an antibody of a phosphorylated peptide produced in situ by PKC. For phospholipase C (PLC) activity, the Amplex^®^ Red Phosphatidylcholine-Specific Phospholipase C Assay Kit (Molecular Probe) was used, which uses the phosphatidylcholine-specific PLC enzyme from *Bacillus cereus* and measures the product through a coupled enzyme assay. For phospholipase A1 (PLA1) activity, the EnzChek Phospholipase A1 Assay Kit (Thermo Fisher Scientific) was used, which uses PLA1 and measures product formation from a fluorescent substrate.

### 4.8. Binding Experiments

Binding experiments were performed by mixing 0.4 mM 1,2-dipalmitoyl-phosphatidylethanolamine (DPPE) or 1,2-dipalmitoyl-phosphatidic acid (DPPA) and 0.4 mM NAI-112 in 2 mM Tris-HCl at pH 7.5 with 8% DMSO. No additional pH modulators were added. After 30 min at room temperature, the binding mixtures were diluted 10 times with 50% acetonitrile and analyzed by direct infusion in low-resolution mass spectrometry in negative and positive ionization modes. The single molecules were separately studied as controls. The instrument was a LCQ-Fleet (Thermo Fisher Scientific, Waltham, MA, USA) equipped with an electrospray interface (ESI) and a tridimensional ion trap. The *m*/*z* ranges were set at 200–2000 with ESI conditions as follows: spray voltage of 3500 V, capillary temperature of 275 °C, sheath gas rate at 15 units, and auxiliary gas rate at 0 units. The flow rate was set at 3 µL/min, and the normalized collision energy used to fragment the complex was 25%.

### 4.9. Selection of Resistant Strains by Direct Plating and Serial Passages

All experiments were performed with *S. aureus* ATCC6538P. The strain was propagated at 37 °C in Mueller–Hinton Broth (BD Difco^TM^) containing 20 mg/L of CaCl_2_ and 10 mg/L of MgCl_2_ (MHBC medium). For selection of resistant strains by direct plating, *S. aureus* cultures were grown until OD_600_ of 0.7, corresponding to 5 × 10^8^ CFU/mL, centrifuged, and resuspended with fresh medium to 1 × 10^10^ CFU/mL. Then, 100 µL were spread on Muller–Hinton Agar (BD Difco^TM^) supplemented with 160 µg/mL or 640 µg/mL of NAI-112 and incubated at 37 °C. Colonies were scored after 10 days. For selection of resistant mutants by serial passages, MHBC medium containing serial, twofold dilutions of NAI-112, starting from 512 µg/mL down to 0.5 µg/mL, was inoculated with 1 × 10^5^ CFU/mL in a 96-well microtiter plate. Cultures were incubated overnight at 37 °C. The culture at the highest concentration that supported growth was diluted to 1 × 10^5^ CFU/mL with fresh medium and added to a fresh plate containing serial dilutions of NAI-112 as before, followed by overnight incubation. This process was continued for 15 passages. From cultures at passage 8 (grown at 64 µg/mL) and passage 15 (grown at 256 µg/mL), single colonies were isolated and named R8.1 and R8.2 (from the eighth series) and R15.1–15.5 (from the fifteenth series).

### 4.10. Antibacterial Assays

MICs were determined by the broth microdilution methodology in sterile 96-well microtiter plates according to Clinical and Laboratory Standards Institute (CLSI) guidelines, as described by Iorio et al. [6]. When indicated, bovine serum albumin was added to MHBC medium at 0.02% (*w*/*v*). Bacteria were inoculated at 1 × 10^5^ CFU/mL, and after 24 h incubation at 37 °C, the MIC was defined as the lowest drug concentration causing complete suppression of visible growth. Growth curves were measured under the same conditions by recording the optical density at 595 nm (OD_595_) over 20 h using a Synergy 2.0 plate reader (BioTeck, Winooski, VT, USA).

### 4.11. Genome Sequencing and Bioinformatic Analysis

Genomic DNA was extracted from the *S. aureus* strains using the GenElute bacterial genomic DNA kit (Sigma-Aldrich, St. Louis, MO, USA) according to the manufacturer’s instructions. Genome sequences were determined by GenProbio Srl (www.genprobio.com, accessed on 23 July 2019) using the Illumina MiSeq platform. DNA libraries were prepared using the Nextera XT DNA sample preparation kit (Illumina, San Diego, CA, USA) according to the manufacturer’s instructions. One ng input DNA from each sample was used for library preparation. The isolated DNA underwent fragmentation, adapter ligation, and amplification. Samples were quantified using Qubit Fluorometer, followed by size evaluation using Tape Station 2200 (Agilent Technologies Santa Clara, CA, USA). The ready-to-go libraries were pooled equimolarly, denatured, and diluted to a sequencing concentration of 15 pM. Library samples were loaded into Flow Cell V3 600 cycles (Illumina) according to the technical support guide. Sequencing cycles resulted in an average reading length of approximately 290 nucleotides for both paired-end sequences.

Fastq files of the paired-end reads were used as input for genome assemblies through the MEGAnnotator pipeline [41]. The SPAdes program version 3.12.0 was used for de novo assembly of the genome sequence [42]. The assembled contigs were ordered using MAUVE software [43], with accession number AP014942.1 as the reference genome. For each sequenced strain, assembly yielded approximately 2.74 Mb of mappable data and resulted in the assembly of 25–31 large contigs, with an average 159–210-fold coverage.

SNPs and INDELs were predicted mapping sequenced reads of each genome against the AP014942.1 GenBank entry as the reference genome. Of note, our sequence of the parental strain ATCC 6538P presented 135 SNPs/INDELs versus the reference genome. These differences were ignored when looking for SNP/INDELs in the mutant strains (Table 2 and Table S1).

### 4.12. Nucleotide Sequence Accession Numbers

The complete genome nucleotide sequences of the wt *S. aureus* ATCC6538P and the resistant strains (R8.1, R15.3, R15.4 and R15.5) were submitted to GenBank under the BioProjects PRJNA766513 and PRJNA764851, respectively.

## Figures and Tables

**Table 1:** MICs (µg/mL) of selected antibiotics against NAI-112-resistant strains.

Antibiotic	Target	MIC (µg/mL)			MIC Ratio (R15.1 to wt)
		wt	R8.1	R15.5	
NAI-112		16	64	128	8
Vancomycin	Lipid II	0.5	1	2	4
NAI-107 *	Lipid II	0.125	0.25	0.25	2
Ramoplanin *	Lipid II	0.06	0.25	0.25	4
Erythromycin	Protein synthesis	0.125	0.125	0.125	1
Ciprofloxacin	DNA replication	0.12	0.12	0.12	1
Rifampicin	Transcription	0.004	0.004	0.004	1

* MICs determined in the presence of 0.02% BSA, as previously reported [16,17].

**Table 2:** Summary of mutations observed in at least two mutants resistant to NAI-112.

Mutant Strain	Genome Position (nt) ^a^	CDS (Locus) ^a^	Type of Mutation	Nucleotide Change	Amino Acid Change	Function
R8.1 R15.3 R15.4 R15.5	27431	SAFDA_0019	SNP	G to A	Cys598Tyr	WalK (two-component sensor histidine kinase)
	587945	SAFDA_0507 to SAFDA_0508	SNP	C to A ^b^	Non-coding	Poly(glycerol-phosphate) alpha-glucosyltransferase –GTP cyclohydrolase
	1340002	SAFDA_1245 to SAFDA_1246	SNP	G to A	Non-coding	Glycine betaine transporter—aconitate hydratase
	1357466	SAFDA_1256	SNP	T to C ^b^	Synonymous	Transposase
	1721972	SAFDA_1584	SNP	A to G	Synonymous	Truncated transposase
	1721999		SNP	T to C	Synonymous	
R8.1 R15.3 R15.5	1722017		SNP	T to C	Synonymous	
	523949	SAFDA_r0010 to SAFDA_0460	SNP	T to G ^b^	Non-coding	5S ribosomal RNA—GntR family transcriptional regulator
R15.3 R15.4 R15.5	1523918	SAFDA_1386	SNP	G to A	Synonymous	Hypothetical protein
	1523955		SNP	A to G	Synonymous	
R8.1 R15.4	1523670		SNP	A to T ^b^	Asp145Glu	
R15.3 R15.5	1523691		SNP	A to T	Asn138Lys	
	1481440	SAFDA_1346 to SAFDA_1347	SNP	A to T	Non-coding	Penicillin-binding protein 2—hypothetical protein
	1481441		DEL	-G		
R15.3 R15.4	512393	SAFDA_0459 to SAFDA_r0004	SNP	A to T	Non-coding	Lysyl-tRNA synthetase—5S ribosomal RNA
R15.4 R15.5	965417	SAFDA_0882 to SAFDA_0883	SNP	C to A ^b^	Non-coding	Hypothetical protein—Na^+^ alanine symporter
R8.1 R15.4	751022	SAFDA_0672	SNP	C to A	Ala451Ser	Di-/tripeptide ABC transporter

^a^ Numbering and annotations are from the reference genome (accession no. AP014942.1). ^b^ Mutation results in a sequence identical to that of the reference genome.

## Data Availability

The genomic sequences were deposited in the NCBI database. Data from lipidome analysis are available from A.A. upon request.

## Supplementary Materials

The following are available online. Figure S1: Inhibition by NAI-112 of protein kinase C and phospolipases A1 and C. Figure S2: (A) MS2 spectrum of the isolated ion *m*/*z* 1507 [M-2H]^2−^. (B) Full mass spectrum of the DPPE–NAI-112 sample in positive ionization mode. The box contains a zoom in the *m*/*z* range 1450–1620. Figure S3: Growth of *S. aureus* ATCC6538P (wt) and of the R8.2 and R15.1–4 mutant strains in the presence of NAI-112. Table S1: Mutations observed in only one mutant strain resistant to NAI-112.

## Sample Availability

Information and requests for resources and reagents should be directed to the corresponding author.
