# Supplementary material for: Understanding the Mechanism of Action of NAI-112, a Lanthipeptide with Potent Antinociceptive Activity
Source: Molecules. 2021 Nov 9;26(22):6764. doi: 10.3390/molecules26226764 (PMC8624038; doi:10.3390/molecules26226764)
Supplement: Supplementary file 1 [file molecules-26-06764-s001.zip › molecules-1419543-supplementary.pdf]

molecules

Supplementary Materials

Understanding the mechanism of action of NAI-112, a lanthipeptide with potent antinociceptive activity

Arianna Tocchetti[1], Marianna Iorio[1,*], Zeeshan Hamid[2], Andrea Armirotti[3], Angelo Reggiani[3] and Stefano Donadio[1]

[1] Naicons Srl, Viale Ortles 22/4, 20139 Milan, Italy
[2] D3 Validation, Fondazione Istituto Italiano di tecnologia, via Morego 30, 16163 Genoa, Italy
[3] Analytical Chemistry Lab, Fondazione Istituto Italiano di tecnologia, via Morego 30, 16163 Genoa, Italy
* Correspondence: miorio@naicons.com

Supplementary results

1. Figure S1;
2. Figure S2;
3. Figure S3;
4. Table S1.

1. Figure S1.

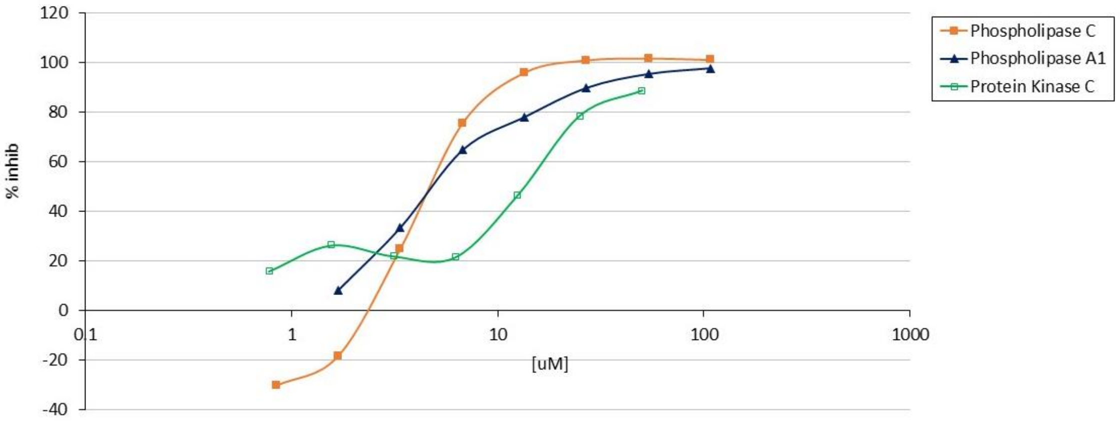

Figure S1. Inhibition by NAI-112 of Protein Kinase C and Phospolipases A1 and C.

2. *Figure S2.*

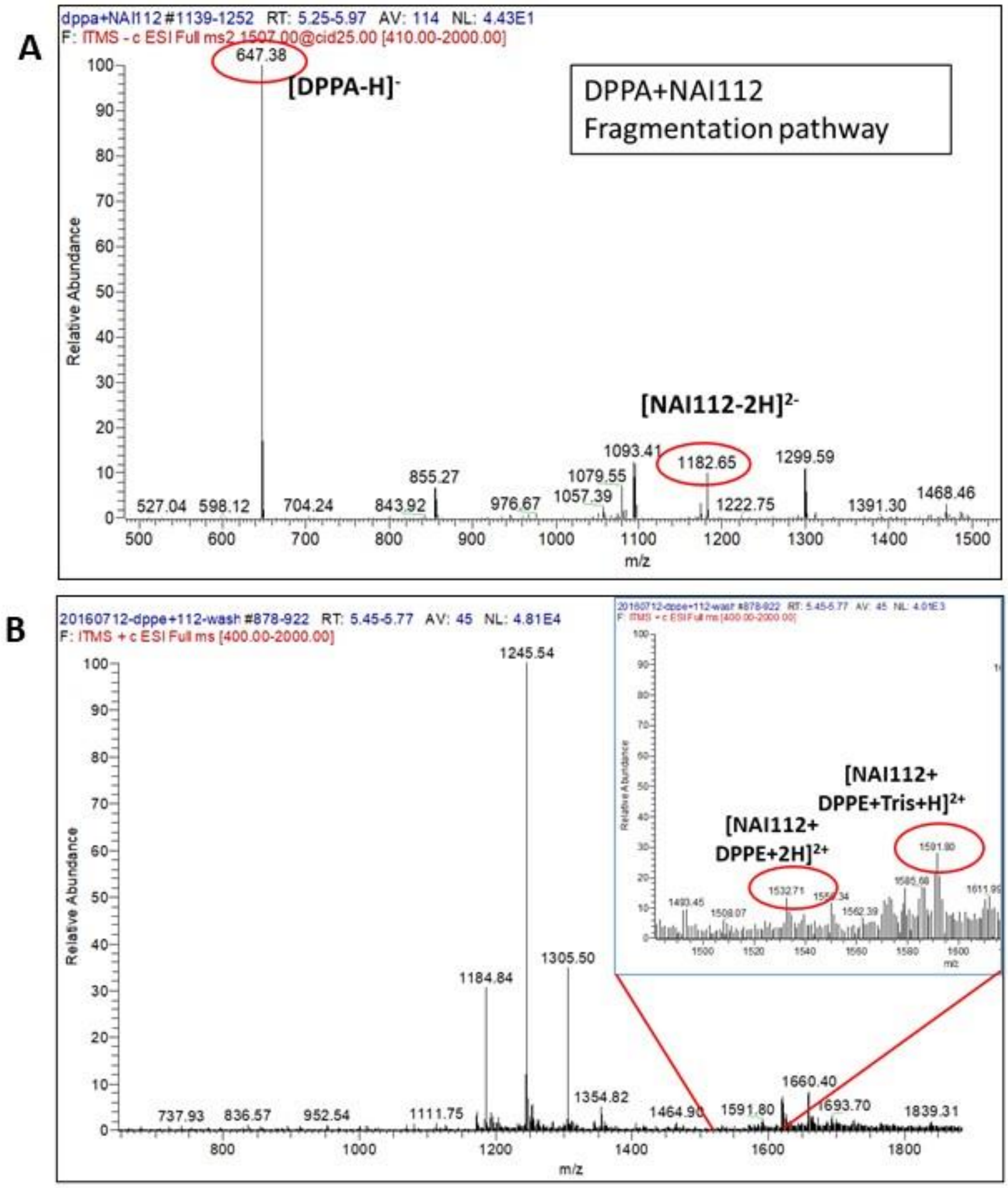

Figure S2. A: MS2 spectrum of the isolated ion m/z 1507 $[M-2H]^{2-}$**.** B: Full mass spectra of "DPPE-NAI112" sample in positive ionization mode. The box contains a zoom in the m/z range 1450-1620.

3. Figure S3.

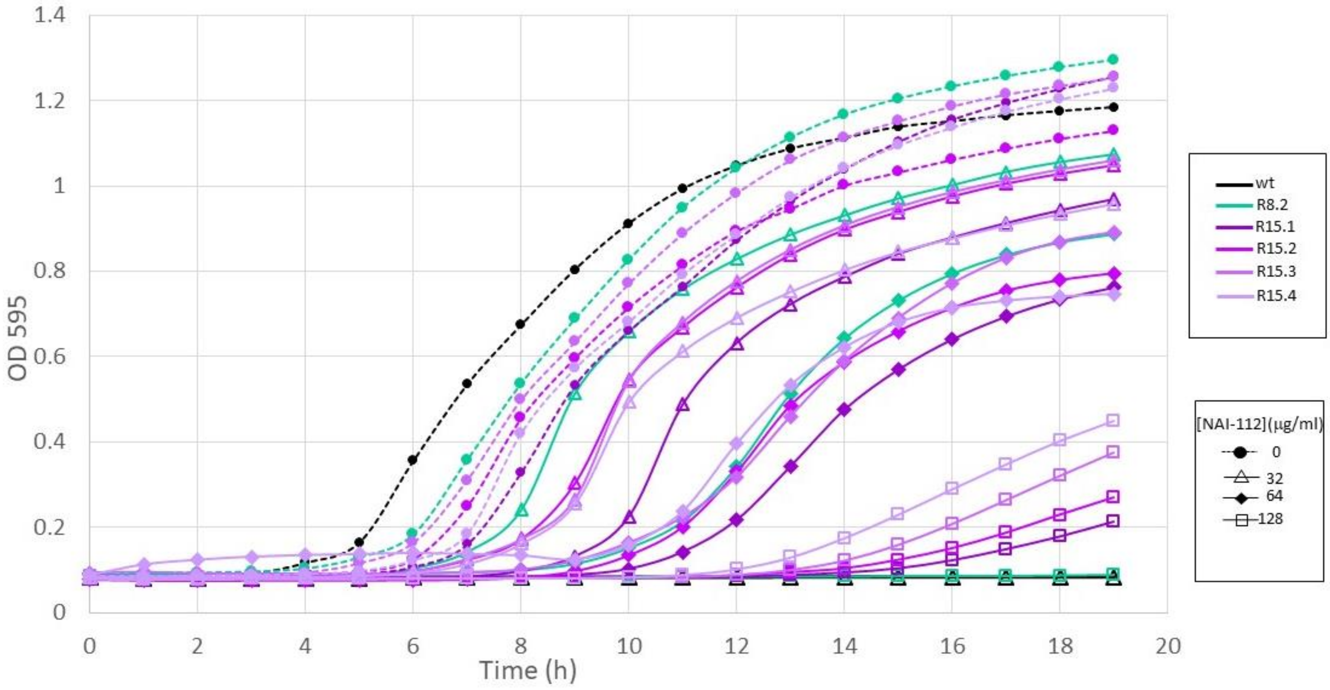

Figure S3. Growth of *S. aureus* ATCC6538P (wt), and of the R8.2 and R15.1-4 mutant strains in the presence of NAI-112.

4. Table S1.

Table S1. Mutations observed in only one mutant strain resistant to NAI-112.

Mutant strain	Genome position (nt) [a]	CDS (locus) [a]	Type of mutation	Nucleotide change	Aminoacid change	Function
R8.1	1650902	SAFDA_1522	DEL	CATCTCTCACTG[b]	del 108-111 (RDAV)	hypothetical protein
	1859248	SAFDA_1703 to SAFDA_1704	DEL	-A	non-coding	serine protease SplA – hypothetical protein
R15.3	731151	SAFDA_0655 to SAFDA_0656	SNP	G to A	non-coding	hypothetical protein – queuosine biosynthesis protein QueE
	1907656	SAFDA_t0034	SNP	G to A		tRNA-Ser
	1907657		SNP	C to G		
	1907666		SNP	G to C		
	2528678	SAFDA_2348	SNP	C to T [b]	Arg410His	surface protein G1
	2612200	SAFDA_2421	SNP	T to C[b]	Glu503Gly	acyltransferase

R15.4	261100	SAFDA_0212 to SAFDA_0213	SNP	G to T [b]	non-coding	cell wall biosynthesis protein ScdA – LytS (two-component sensor histidine kinase)
	261127		SNP	G to A [b]		
	984361	SAFDA_0899	SNP	A to G [b]	Lys45Glu	hypothetical protein
	1923796	SAFDA_1757	SNP	G to A [b]	Gly10Glu	hypothetical protein
	2535962	SAFDA_2353	SNP	G to T [b]	Thr26Asn	hypothetical protein
R15.5	2287402	SAFDA_2121 to SAFDA_2122	SNP	T to C [b]	non-coding	between truncated transposases
R15.3	512392	SAFDA_0459 to SAFDA_r0004	SNP	C to A	non-coding	lysyl-tRNA synthetase – 5S ribosomal RNA
	512395		SNP	A to T		
	512388		SNP	C to A		
R15.3	523914	SAFDA_r0010 to SAFDA_0460	SNP	A to G [b]	non-coding	5S ribosomal RNA – GntR family transcriptional regulator
R15.4	523941		DEL	-G [b]		

[a] numbering and annotations are from reference genome (accession number AP014942.1).

[b] mutation results in sequence identical to that of reference genome.
